# Exploration of the Inhibitory Potential of Varespladib for Snakebite Envenomation

**DOI:** 10.3390/molecules23020391

**Published:** 2018-02-12

**Authors:** Yiding Wang, Jing Zhang, Denghong Zhang, Huixiang Xiao, Shengwei Xiong, Chunhong Huang

**Affiliations:** School of Basic Medical Sciences, Nanchang University, Nanchang 330006, China

**Keywords:** antivenom, myotoxicity, phospholipase A_2_, varespladib

## Abstract

Phospholipase A_2_s (PLA_2_) is a major component of snake venom with diverse pathologic toxicities and, therefore, a potential target for antivenom therapy. Varespladib was initially designed as an inhibitor of mammal PLA_2_s, and was recently repurposed to a broad-spectrum inhibitor of PLA_2_ in snake venom. To evaluate the protective abilities of varespladib to hemorrhage, myonecrosis, and systemic toxicities that are inflicted by different crude snake venoms, subcutaneous ecchymosis, muscle damage, and biochemical variation in serum enzymes derived from the envenomed mice were determined, respectively. Varespladib treatment showed a significant inhibitory effect to snake venom PLA_2_, which was estimated by IC_50_ in vitro and ED_50_ in vivo. In animal models, the severely hemorrhagic toxicity of *D. acutus* and *A. halys* venom was almost fully inhibited after administration of varespladib. Moreover, signs of edema in gastrocnemius muscle were remarkably attenuated by administration of varespladib, with a reduced loss of myonecrosis and desmin. Serum levels of creatine kinase, lactate dehydrogenase isoenzyme 1, aspartate transaminase, and alanine transaminase were down-regulated after treatment with varespladib, which indicated the protection to viscera injury. In conclusion, varespladib may be a potential first-line drug candidate in snakebite envenomation first aid or clinical therapy.

## 1. Introduction

It is estimated that about 2.5 million envenomation cases occur around the world, resulting in 100,000 deaths annually [1,2]. However, an effective treatment regimen to treat lethal snakebite envenomation remains elusive due to insufficient attention. The geographical imbalance of snakebite incidence and local antivenom production or supply remains a significant issue in those disadvantaged rural communities of tropical and sub-tropical developing countries in sub-Saharan Africa, Asia, Latin America, and parts of Oceania. Furthermore, categories of antivenoms are far fewer than snake species of medical importance. For instance, only four antivenoms specific to *Deinagkistrodon acutus*, *Agkistrodon halys*, *Bungarus multicinctus*, and *Naja atra* are manufactured in China, whereas there are about 50 species of venomous snakes. To complicate matters, some marketed antivenoms appear clinically ineffective [3,4]. Snakebite envenomation was added to the priority list of neglected tropical diseases (NTDs) by the World Health Organization (WHO) in 2017, calling for increased scientific investigation and financial input [5].

Since most snakebite envenomations are acute and often occur in rural or tropical areas where clinical service is insufficient, without proper treatment, patients may suffer severe irreversible myonecrosis or neural paralysis, consequently causing high morbidity and mortality [2]. Snakebite envenomation often causes severe local damage, including edema, hemorrhage, myonecrosis, and systemic toxic response, including organ failure and coagulopathy [6,7,8]. Myonecrosis and hemorrhage are the most devastating consequences of *Viperidae* envenomation, whereas neural paralysis is a lethal threat of *Elapidae* attacks [9,10].

Traditionally, snake envenomation can be well neutralized by antivenin derived from immunized animals. However, multiclonal antibodies are highly specific to snake venom, which indicates that correct identification of snakebites is of utmost importance for correct antivenin application [11]. Although antivenom is the mainstay of primary treatment for snake envenomation, there remain limitations in safety, efficacy, cold storage conditions, and economic aspects in manufacturing. Serum sickness, including fever, urticaria, nausea, tachycardia, arthralgia, and hypotension, could occur immediately after administration with a high incidence of 6–23% [12]. Moreover, due to the non-profitable nature, the drug company Sanofi Pasteur in Lyon, France ceased the production of Fav-Afrique, a highly-effective antivenin, in 2014, resulting in a worldwide snakebite crisis [13]. Several alternatives have been proposed, which were rarely qualified as a clinical treatment. These included acetylcholinesterase-inhibiting drugs (neostigmine, edrophonium), which were thought to reduce the neuromuscular block from neurotoxic snakebites by increasing the level of acetylcholine at neuromuscular junctions [14]. Snake venom metalloproteinases (svMP) and serine protease (SP) inhibitors were also considered as potential solutions to snakebites. Nevertheless, the use of svMP and SP to treat snakebites is still controversial [5,15,16]. The demand of first-line anti-venom drugs that are portable, universal, and highly effective is extremely urgent. However, due to the component-complexity of snake venom, broad-spectrum inhibitors are currently still under investigation.

Phospholipase A_2_(PLA_2_) is a type of enzyme that catalyzes the hydrolysis of the glycerol phosphate sn-2 acyl ester bond in lipoproteins and the cell membrane [16]. Snake venom PLA_2_ (svPLA_2_) is abundant in all venomous families. Currently, 383 svPLA_2_s from 103 snake species are listed in the Expasy protein database. SvPLA_2_s have been shown to have a variety of toxic effects, including bleeding [17], myonecrosis [18], neuromuscular paralysis [19], platelet aggregation [20], edema, and a wide-spread inflammatory response [21]. Thus, as a common and devastating component in snake venom, svPLA_2_s represent promising targets for broad-spectrum and effective anti-venom drug development. In a previous study, Garcia et al. used a non-toxic PLA_2_ derived from *B. alternatus* venom as an antigen to immunize rabbits, and applied the resulting anti-PLA_2_ IgG to mice that were injected with *B. alternates* venom. The hemorrhagic and myotoxic effects were completely inhibited by anti-PLA_2_ IgG, and all mice survived over 48 h after they were administered four times the LD_50_ dose venom and anti-PLA_2_ IgG [22].

Veraspladib (LY315920) is an effective pan-inhibitor of mammalian secretory phospholipase A_2_s (sPLA_2_s) and designed as an anti-inflammatory drug candidate by disrupting arachidonic acid release from glycerolipids [23]. In 2016, it was repurposed as a nonspecific inhibitor of svPLA_2_s by Lewin et al. Varespladib, and its orally bioavailable prodrug methyl-varespladib (LY333013), showed strong inhibition of 28 types of svPLA_2_s from six continents [24]. It also exerted beneficial effects on mice that were inoculated with eastern coral snake (*Micrurus fulvius*) venom, which was considered to have the highest sPLA_2_ activity and most intense hemo- and neurotoxic effects [24]. However, the effects of varespladib on different crude venoms in vitro, as well as the overall protection to local damage and the systemic response of envenomation in vivo are under-evaluated. In this study, we determined the inhibitory effects of varespladib on PLA_2_ activities, hemorrhage, myonecrosis, and viscera damage inflicted by four categories of Chinese snake venom, including *D. acutus*, *A. halys*, *B. multicinctus*, and *N. atra*. Due to the disadvantages of antivenin, we hope that further investigation will facilitate the drug development of varespladib to be a broad-spectrum antivenom.

## 2. Results

### 2.1. Inhibition of Phospholipase A_2_ In Vitro

**Figure 1:**
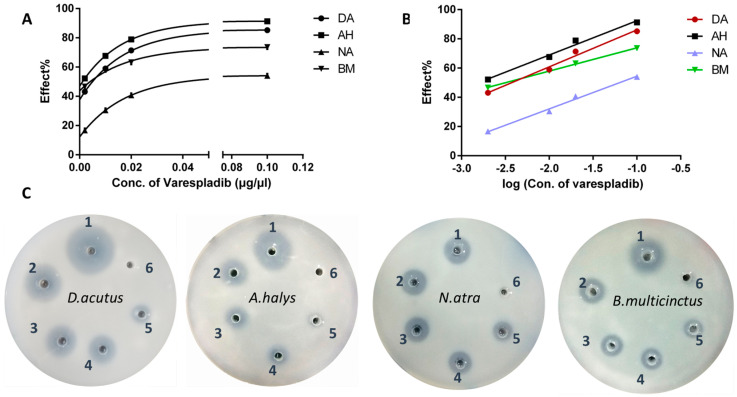
The anti-catalytic effect of varespladib on snake venom phospholipase A_2_ in vitro. (**A**) The varespladib concentration versus the inhibitory effect (cut-down area of varespladib group/venom alone); (**B**) the inhibitory effect was linearly proportional to the logarithm of varespladib concentration; and (**C**) the anti-catalytic activities of varespladib to *D. acutus*, *A. halys*, *N. atra*, and *B. multicinctus*. Well 1 was the positive control, filled with venom alone, well 2–5 were mixtures of venom and 0.1, 0.5, 1, and 5 μg varespladib. Abbreviations: DA: *D. acutus*, AH: *A. halys*, NA: *N. atra*, and BM: *B. multicinctus*.

Based on the area of transparent circles, the digestion of egg yolk lecithin differed in the four different snake venoms (Figure 1). Hemotoxic venom derived from *D. acutus* and *A. halys* had higher PLA_2_ activities compared to neurotoxic venom from *N. atra* and *B. multicinctus.* The vehicle (20% DMSO) had no effects on lecithin cleavage, however, varespladib had a dose-dependent inhibitory effect on svPLA2’s enzymatic activities. The logarithm of varespladib concentration was linearly proportional to the inhibitory effect (∆transparent cycle area/area in positive control group) (Figure 1A,B). The equation and IC_50_ of varespladib to the four venoms were as follows:

*D. acutus*, Y = 25.28X + 111.4, R^2^ = 0.9865; IC_50_ = 0.0037 μg/μL
*A. halys*, AH: Y = 23.47X + 115.9, R^2^ = 0.9867; IC_50_ = 0.0016 μg/μL
*N. atra*, Y = 22.36X + 76.82, R^2^ = 0.9916; IC_50_ = 0.063 μg/μL
*B. multicinctus*, Y = 15.92X + 89.80, R^2^ = 0.9991. IC_50_ = 0.0032 μg/μL

### 2.2. Anti-Hemorrhage Effect In Vivo

**Figure 2:**
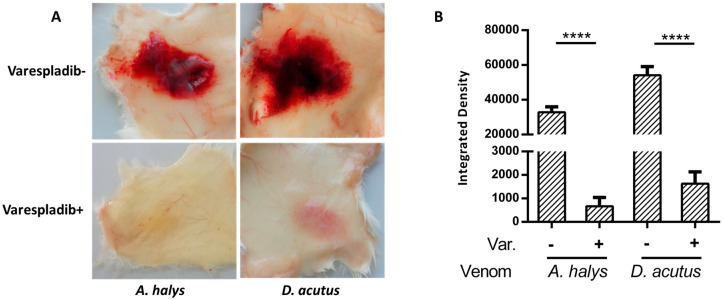
Inhibitory effect of varespladib on hemorrhagic toxicity. (**A**) Severe hemorrhage and ulceration were caused by subcutaneous injection of *A. halys* and *D. acutus* venom, in contrast to varespladib-treated mice that had intact blood vessels and showed mild symptoms; and (**B**) varespladib significantly reduced the integrated density of hemorrhagic plaques (**** *p* < 0.0001).

Venom of Viperidae are known to possess strong hemorrhagic toxicities. Mice that were inoculated with *D. acutus* venom generated obvious hemorrhagic plaque (33.10 × 22.30 mm), whereas petechiae caused by venom derived from *A. halys* were 24.91 × 17.93 mm. Due to the intense toxicity, mice also exhibited severe damage to blood vessels and ulceration. In contrast, mice in the varespladib group exhibited mild swelling and yellow exudate around the injection area with intact blood vessels, and no hemorrhagic spots were observed (Figure 2). The integrated density of hemorrhagic plaques in varespladib-treated mice were significantly lower compared to positive groups, indicating the strong inhibitory ability of varespladib towards snake venom.

### 2.3. Physiological and Behavioral Signs

The muscle envenomed mice showed blunt responses and torpid movement. They were hunched, somnolent with floppy heads, and occasionally licked the envenomation site, which may indicate they were in pain. Moreover, signs of protopsis and whitening eyeballs were distinct in the *N. atra* and *D. acutus* groups, whereas antemortem spasms and convulsion were observed in mice in the *N. atra* and *B. multicinctus* groups. Two mice in the *N. atra* and *B. multicinctus* venom group died within 4 h after the venom injection due to respiratory paralysis. However, all varespladib-injected mice were alive until the end of experiment. The overall physiological and behavioral signs were near normal and mice were vibrant in their drinking, eating, and movement behavior.

### 2.4. Local Reaction in the Gastrocnemius Muscle

**Figure 3:**
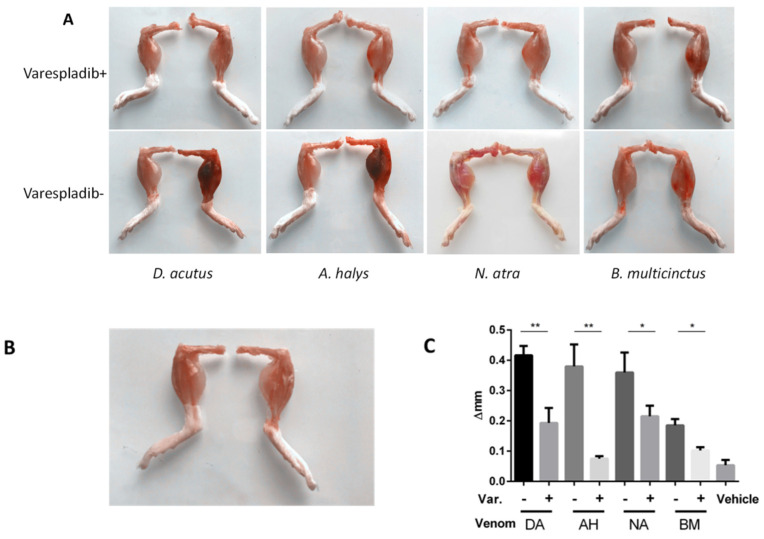
Myonecrosis in the gastrocnemius muscle. (**A**) Mice injected with venom derived from *D. acutus* and *A. halys* suffered from severe hemorrhage and edema in the envenomed leg. Moreover, the gastrocnemius muscles near the injection site had a deep red color and showed necrosis, swelling, and erosion of the muscular fasciae. When compared with the non-injected leg from the same mouse, the gastrocnemius of varespladib plus mice was normal in color and size; (**B**) negative control mice, injected with 20% DMSO; and (**C**) In all venom groups, the level of edema was significantly higher when compared to the corresponding varespladib inhibitory groups. ∆mm represents the difference in the width of the left limb gastrocnemius compared to the contralateral muscle. * *p* < 0.05, ** *p* < 0.01 (*n* = 3). Abbreviations: DA: *D. acutus*, AH: *A. halys*, NA: *N. atra*, and BM: *B. multicinctus*.

The mice injected with venom derived from *D. acutus* and *A. halys* suffered severe hemorrhage in the envenomed leg, and the inoculated gastrocnemius muscles exhibited a series of envenomation alternatives, including a deep red color, swelling, necrosis, and erosion of the muscular fasciae (Figure 3A,B). In contrast, the envenomed gastrocnemius of varespladib mice appeared normal, both in color and size, when compared with the contralateral leg. Obvious swelling was observed in gastrocnemius inoculated with venom derived from *N. atra* venom and *B. multicinctus*, while hemorrhage was inconspicuous. A total reduction in edema of 62%, 81%, 31%, and 38% was achieved in varespladib plus groups of *D. acutus*, *A. halys*, *N. atra*, and *B. multicinctus*, respectively (Figure 3C). Thus, varespladib was powerful not only in controlling hemorrhagic toxicity, but also in inhibiting the edematous response.

### 2.5. Immunohistochemistry and Desmin Loss

**Figure 4:**
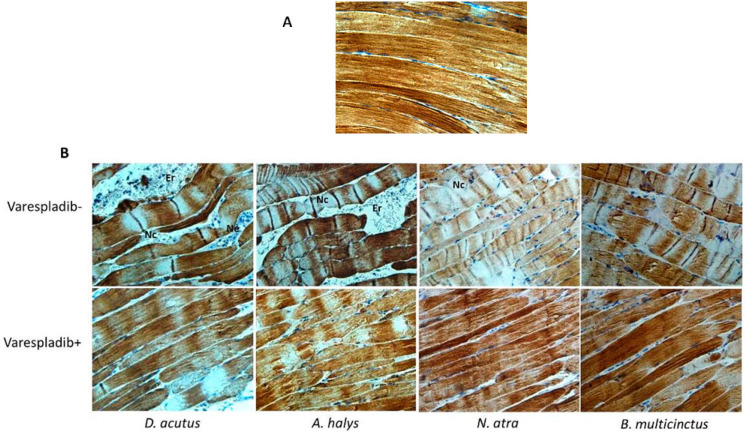
Immunohistochemical staining of desmin in gastrocnemius muscle. (**A**) Negative control inoculated with 50 μL of 20% DMSO, the staining was intense in color and evenly distributed at Z lines. The muscle fibers were integrated and arranged in a normal structure; and (**B**) the muscle fibers were arranged chaotically, and evident myonecrosis was observed both in *A. halys* and *D. acutus* groups. Noticeably, massive infiltration of erythrocytes and inflammatory cells was found in the intramuscular space. The desmin content in all envenomed groups suffered sectional loss, whereas the loss in varespladib plus groups was less. Muscle sections were examined at a magnification of 400×. Abbreviations: Nc, necrosis; Ne, neutrophil.

The pathological changes observed in gastrocnemius muscle of envenomed mice were examined by immunohistochemistry. The vehicle group showed bunchy muscle fibers, intact perimysium, and very few inflammatory cell infiltrations. However, obvious swollen and distorted myofibers, myofilament contraction, and desmin degradation were observed in gastrocnemius muscles of all envenomed mice. An expanded myofiber gap, which resulted in infiltration of a large number of erythrocytes and neutrophilic granulocytes were found in mice that were envenomed with venom derived from *D. acutus* and *A. halys*, which may be the reason of the edema observed in the gastrocnemius muscle. Venom of *N. atra* and *B. multicinctus* also caused endomysium erosion, and swollen myofibers and local edema were observed in the envenomed limb. In contrast, erythrocyte infiltration was weak in the neurotoxic venom group, hence, less hemorrhage was observed. The desmin concentration and distribution in these envenomed mice was significant lower and uneven when compared to the negative control and varespladib-treated groups. Moreover, the architectural organization of gastrocnemius muscle in varespladib plus mice were less disrupted (Figure 4B). Myocytes and the endomysium remained intact, and except for occasional signs of myonecrosis, infiltration of erythrocytes and neutrophils were rarely seen in the presence of varespladib.

**Figure 5:**
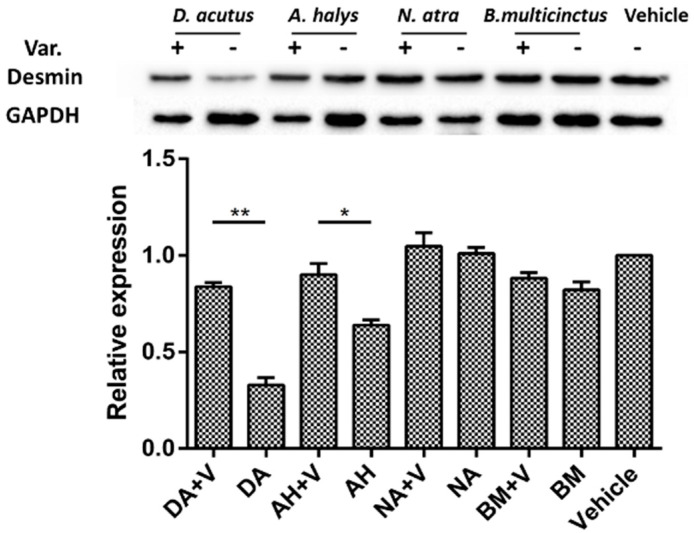
Desmin loss in gastrocnemius muscle exposed to different types of venom. Desmin was obviously degraded in gastrocnemius muscles from envenomed mice, especially venom from *D. acutus* and *A. halys* groups. However, the desmin content only slightly decreased in *N. atra* and *B. multicinctus* groups. Nevertheless, desmin loss was significantly relieved in the presence of varespladib. * *p* < 0.05, ** *p* < 0.01. Abbreviations: DA: *D. acutus*, AH: *A. halys*, NA: *N. atra*, and BM: *B. multicinctus*.

Desmin degradation was also evidenced by Western blot analysis. Significant loss was observed in mice that were injected with venom derived from *D. acutus* and *A. halys*. The inhibition of varespladib on desmin degradation in the two hemotoxic venom groups was remarkable. A similar tendency was found in *N. atra* and *B. multicinctus* groups, but the inhibition of desmin loss was less significant (Figure 5).

### 2.6. Biochemical Assays of Serum CK, LDH1, AST, and ALT Levels

As a sensitive biomarker of skeletal muscle damage, serum CK levels elevated significantly in envenomed mice due to myonecrosis. In addition, dramatic decreases were achieved in varespladib plus groups, except for the *B. multicinctus* group, in which the CK value slightly fluctuated. The CK level positively correlated with the severity of muscle degeneration and necrosis observed by histological analysis.

**Figure 6:**
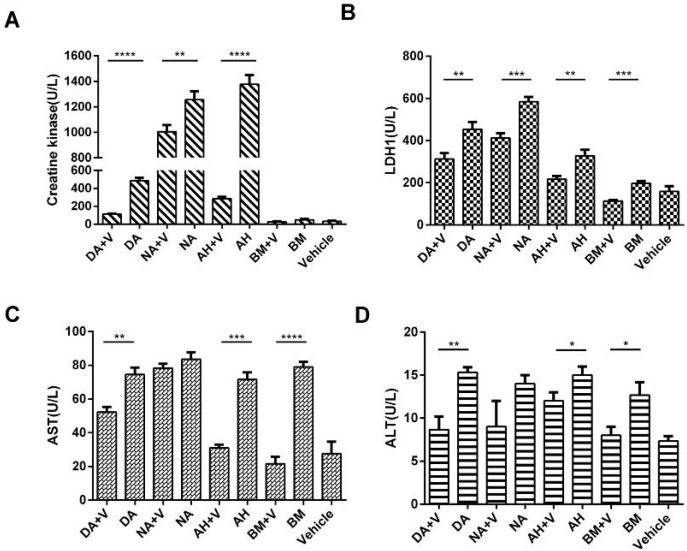
Alterations in serum CK, LDH1, AST, and ALT levels following snake envenomation. (**A**) Serum CK level in DA, NA and AH envenomed mice significantly increased, while it dramatically reduced in the presence of varespladib; (**B**) Serum LDH1 in all envenomed group showed overtly higher activity than varespladib group; (**C**,**D**) AST and ALT levels exhibited remarkable increase in DA, AH and BM envenomed group, but the effect was significantly relieved with varespladib. The systemic damage following envenomation was dramatically reduced in the presence of varespladib. * *p* < 0.05, ** *p* < 0.01, *** *p* < 0.001, **** *p* < 0.0001.

Serum LDH1, AST, and ALT levels were also assayed to evaluate the systemic damage following envenomation in the viscera, in particular in the liver and heart. LDH1 and AST levels were elevated in the four envenomed groups, particularly in *D. acutus*, *A. halys*, and *N. atra*, indicating increased damage in the myocardium. ALT levels were also increased in envenomed mice but were still within the normal range in which slight hepatic impairment may have occurred. In contrast, the liberation of LDH1, AST, and ALT levels were significantly attenuated by varespladib (Figure 6). Obviously, varespladib exhibited powerful inhibitory effects on the systemic toxic response induced by the different types of venom.

### 2.7. The Estimation of the Median Effective Dose (ED_50_) of Varespladib

The lethal toxicities of Viperid were less than Elapid venoms, although the mice injected with a high dose of *D. acutus* and *A. halys* venom exhibited overt abdominal edema and hemorrhage on necropsy. Mice inoculated with 4 × LD_50_ dose of *B. Multicinctus* and *N. atra* usually died within 2 h with similar flaccid paralysis. However, varespladib was more effective to rescue the envenomed mice of Viperid venom than that of Elapid (Table 1).

## 3. Discussion

Due to its severe toxicity and unavailability of proper drugs, snakebites were thought to be an ignorant global public health conundrum that leads to high mortality and permanent disability [2]. The PLA_2_ family has recently been regarded a promising target for broad spectrum antivenom drug development, due to their wide-distribution, high content, and diverse toxicological effects [25]. Previous studies revealed that PLA_2_s acts with other toxic components in a synergistic manner, mostly zinc-dependent metalloproteinases, which are responsible for hemorrhage, blistering, and necrosis [26,27,28]. The anti-PLA_2_ IgGs developed from Ba spII RP4 PLA_2_ (a non-toxic PLA_2_ from *Bothrops alternates* venom) could inhibit haemolysis and myotoxicity of the crude venom, and also decreased 50% of mortality of mice dosed with four times the LD_50_. The protection effect of the anti-PLA_2_ IgGs suggested Ba SpII RP4 PLA2 had a synergistic effect on whole-venom toxicity [22]. Therefore, PLA_2_ became a valuable target in solving snakebite envenomation.

The in vitro agar plate assay demonstrated that varespladib was extensive and effective in blocking the PLA_2_ catalytic activity of the four types of snake venom, which was in accordance with the findings presented by Lewin et al. when using chromogenic assays [24]. However, varespladib was more effective to venom derived from Viperidae compared to that from Elapidae. It is well-known that svPLA_2_s can be divided into catalytic Asp49- and non-catalytic Lys49 subtypes. Lys49-PLA_2_ (also named group I) are mainly distributed in snake venom of Elapidae, thus, the lower efficiency to *N. atra* venom may be due to a weaker binding affinity of varespladib to Lys49-PLA_2_ subtypes. The survival benefit of varespladib to *B. multicinctus* might be due to the inhibition to β-bungarotoxins, a dimer consisting of a IA-type PLA_2_ subunit and a disulfide-linked BPTI-like peptide. Nevertheless, this hypothesis would need to be verified by additional studies. Similarly, the proof-of-concept (in vivo) tests revealed that varespladib strongly inhibited the toxicity of four venoms, however, a higher dose was needed in elapid venoms. Other labs also reported that varespladib can extend survival against *C. atrox*, *C. scutulatus*, and *D. russelli*, which suggests potency and possibility of varespladib in the pre-referral therapy against the broad-spectrum envenomation [24].

In this study, the hemorrhagic toxicity triggered by *D. acutus* and *A. halys* was nearly completely inhibited by varespladib when administered subcutaneously or intramuscularly. As a small chemical inhibitor initially designed for mammal secretory PLA_2_s, varespladib also decreased the release of CK, LDH1, AST, and ALT in all envenomed mice. The elevation of these serum biomarkers of local injury and visceral impairment are generally the result of a combined action of snake venom components. It was evidential that PLA_2_s synergistically act with other venom components, therefore PLA_2_s may play a central role in the toxicity of snake venoms.

Venom derived from *D. acutus* and *A. halys* were also myotoxic, and caused 52% and 31% desmin loss, respectively. Desmin degradation was previously reported in *Notechis scutatus* snake envenomation [29], and could be attenuated by snake endogenous PLIγ (gamma type PLA2 inhibitor protein) [28]. The breakdown of desmin was further demonstrated by immunohistological evaluation. In muscle fibers, desmin formed a scaffold around the Z-disk of the sarcomere [30], connected the Z-disk to the subsarcolemmal cytoskeleton, and laterally linked myofibrils, thereby maintaining the structural and mechanical integrity of the cell during contraction. Sarcomere contraction and myofiber necrosis was observed in *D. acutus* and *A. halys* envenomed mice, and the destruction was hampered in the presence of varespladib. In muscle fibers of all envenomed muscle, significant signs of infiltration were observed, which were significantly reduced in varespladib-treated groups. A synchronous inhibition to mouse PLA_2_s might be exerted by varespladib to prevent the activation of the arachidonic acid inflammation pathway.

Varespladib was more likely potent in anti-Viperid venoms than that of Elapid. The ED_50_ against *D. acutus* and *A. halys* was 1.14 and 0.45 μg/g, whereas the ED_50_ value were up to 15–22-fold greater in *B. multicinctus* and *N. atra* envenomed mice. The reason was probably due to the lower PLA_2_ content in the two Elapid venoms [25].

Our study showed that varespladib, as a broad-spectrum inhibitor of svPLA_2_ with a low IC_50_, exhibited significant inhibitory effects on pathological alternatives caused by snake venom. In addition, the clinical phase II trials suggested that six months of varespladib therapy by 500 mg daily in acute coronary syndrome (ACS) patients generated indistinctive adverse cardiovascular events from the placebo population [31]. The dosage of varespladib to an envenomed patient, for instance, an adult man of 70 kg, will be 280 mg based on the pilot trial. Thus, varespladib is favorable both in efficacy and safety for snakebite envenomation treatment. Due to its good characteristics, including high absorbability, low-cost, thermostability, and potency, varespladib may serve as a prominent pre-referral intervention, and provide a timely first-aid possibility to avoid permanent sequelae or death caused by acute envenomation.

## 4. Materials and Methods

### 4.1. Reagents and Materials

Snake venom lyophilized powder of *D. acutus*, *A. halys*, *B. multicinctus*, and *N. atra* were obtained from the Huangshan Snake Farm (HuangShan, China). Varespladib was purchased from Jiyi Pharmatech Company (Shanghai, China) and dissolved in 20% dimethyl sulfoxide (DMSO). Rabbit anti-desmin monoclonal antibody (ab32362) was from Abcam Biotech Company (Cambridge, UK). Mouse anti-GAPDH monoclonal antibody (TA505454), anti-rabbit IgG secondary antibody (ZB-2301), and anti-mouse IgG secondary antibody (ZB-2305) were from Zsbio Biological (Beijing, China). Clarity™ ECL Western blot substrate was from Bio-Rad (Hercules, CA, USA). Assay kits for CK (A032), LDH1 (A020-3), ALT (C009-2), and AST (C010-1) were purchased from Nanjing Jiancheng Bioengineering Institute (Nanjing, China).

Electrophoresis chambers, power supply, and gel imaging system were purchased from Bio-Rad (Hercules, CA, USA). Tissue embedding machine and histotome were from Labsun (Karslruhe, Germany).

### 4.2. Inhibition of Varespladib on svPLA_2_ In Vitro

A modified egg yolk agar (EYA) plate method was performed to evaluate the inhibitory effect of varespladib on svPLA_2_ activity as described previously [32]. In brief, an egg yolk suspension was pre-mixed with CaCl_2_ solution, poured into culture dishes and immediately six circular wells (diameter = 5 mm) were punched into the agar. Snake venom solution mixed with different amounts of varespladib was added into wells. Well 1 was filled with 50 μL of *D. acutus* venom (40 μg) as a positive control, whereas to well 2–5 a mixture of *D. acutus* venom, containing 0.1, 0.5, 1.0, or 5.0 μg of varespladib was added. Well 6 contained a similar volume of normal saline and served as a negative control. Parallel assays with equal amounts (40 μg) of *A. halys*, *B. multicinctus*, and *N. atra* venom were conducted in a similar fashion. The EYA plates were incubated at 37 °C for 6 h, and the inhibitory effect of varespladib on svPLA_2_ was assessed based on the area ratio (cut-down area of varespladib group/well 1) of transparent circles. GraphPad Prism 6.01 (La Jolla, CA, USA) software was used to obtain the equation which was based on the logarithm of PLA_2_ dosage and area ratio. Half maximal inhibitory concentration (IC_50_) was defined as the concentration of varespladib that resulted in half of the transparent circle area in venom only well. The IC_50_ of varespladib to the four venoms were calculated based on the corresponding equations.

### 4.3. Anti-Hemorrhage Effect In Vivo

Female Kunming mice weighing between 20 ± 2 g at 12 weeks of age were obtained from the Animal center of Nanchang University (Nanchang, China). Mice were acclimatized for five days under standard laboratory conditions of diet, water, and temperature. The animal experiments were carried out in accordance with the guidelines issued by the Ethical Committee of Nanchang University (NDSYDWLL-201761).

Mice were randomly divided into four groups (*n* = 3 mice/group). Mice in the venom groups were subcutaneously inoculated with 50 μL of 2 × LD_50_ dosage of *D. acutus* (80 μg) or *A. halys* (40 μg) venom at the centrodorsal position. Mice in the treated groups were administered with a similar dosage of venom, which was premixed with 4 mg/kg of varespladib [24]. Three hours later, mice were euthanized by cervical dislocation and skinned to estimate the anti-hemorrhage effect. Image J software (National Institutes of Health, Bethesda, MD, USA) was used to assess the inhibition on hemorrhage by the integrated density of hemorrhagic plaques on the inner skin.

### 4.4. Inhibition to Myotoxicity

Mice were randomly assigned to nine groups (3 mice/group). As in the anti-hemorrhage experiment described in Section 4.3, 50 μL of snake venom or a mixture of venom with varespladib was inoculated in the middle of the gastrocnemius muscle of the right limb. Based on a study described by Liu et al. [33], the dosage of snake venom used was 2 × LD_50_ of *D. acutus* (80 μg), *A. halys* (40 μg), *B. multicinctus* (12 μg), and *N. atra* (28 μg). The amount of varespladib was 4 mg/kg of body weight. The varespladib vehicle (20% DMSO) was used as the negative control.

Six hours after the injection, mice were sacrificed by cervical dislocation and blood was immediately taken by cardiac puncture and collected into heparin-coated tubes. Serum were obtained by centrifugation at 12,000 rpm for 15 min and assayed to determine the levels of CK, LDH1, and AST according to the guidelines provided with the kits.

Per mouse, the gastrocnemius of both hind limbs was exposed by carefully removing the surrounding muscles. The gastrocnemius muscle was prepared in the same fashion and measured by a Vernier caliper. Delta width was calculated by subtraction of the right limb (envenomed) to the contralateral one (left, non-injected). Differences in width between the venom groups and the varespladib-treated groups were analyzed to evaluate if the inhibitory effect to local edema was significant.

#### 4.4.1. Desmin Degradation in Envenomed Gastrocnemius Muscle

The gastrocnemius muscle of right limb was homogenized using an electric homogenizer in cold RIPA buffer with fresh phenylmethylsulfonyl fluoride (PMSF). After centrifugation for 5 min at 12,000 rpm at 4 °C, the supernatant was collected and the total protein concentration was determined by bicinchoninic acid (BCA) assay (Thermo Fisher Scientific, Waltham, MA, USA). Protein samples were mixed with loading buffer, denatured by boiling for 5 min, and loaded on a 12% polyacrylamide gels at an equal protein amount of 40 μg per well. Proteins were separated by SDS-PAGE and electrotransferred to polyvinylidene fluoride (PVDF) membranes according to a regular protocol. Subsequently, membranes were blocked and cut into two strips at the position of 45 kDa. The upper and lower membrane strips were incubated overnight with a monoclonal anti-desmin antibody (1:5000) or anti-GAPDH antibody (1:2000), respectively. Next, membranes were rinsed and incubated with anti-rabbit or anti-mouse IgG-horseradish peroxidase (HRP)-conjugated secondary antibody, respectively. Desmin and GAPDH-related protein bands were illuminated by ECL reagent and captured by a gel imaging system. Image J software was used to analyze the intensity of the bands.

#### 4.4.2. Immunohistochemical Analysis

Gastrocnemius specimens were fixed for 24 h in 4% paraformaldehyde and dehydrated in ascending grades of alcohol. Specimens were embedded in paraffin, sliced longitudinally into 4 μm-thick sections and mounted on glass slides. Tissue sections were deparaffinized in xylene and rehydrated through descending alcohols. Then, sections were microwaved in citrate-based buffer for antigen retrieval as per conventional methods. Sections were incubated overnight at 4 °C with a rabbit anti-desmin monoclonal antibody at a 1000-fold dilution. The reaction was developed using anti-rabbit IgG horseradish peroxidase (HRP)-conjugated secondary antibody (1:500) for 30 min. Sections were incubated with diaminobenzidine (DAB) for 5 min for visualization, and counterstained with hematoxylin for 10 min. The architecture of gastrocnemius muscle as well as desmin expression were observed on an Olympus biological microscope system (Tokyo, Japan)

### 4.5. Estimation of the Median Effective Dose (ED_50_) of Varespladib against Snake Venoms

To evaluate the dose effect of varespladib in survival improvement of envenomed mice, five groups of mice (*n* = 10 per group) for each snake venom were set and challenged to the mixture of high-dose snake venom and a gradient amount of varepladib. The challenge dose was determined based on a pilot test; in brief, *D. acutus* and *A. halys* groups were dosed with six-fold LD_50_, while *B. multicinctus* and *N. atra* groups were treated with two-fold LD_50_. The varespladib dose used in *D. acutus* groups was 0.16–2.56 μg/g body weight in a two-fold gradient. The gradient in other venoms were 1.7-fold, namely, a dose from 0.394 to 3.292 μg/g in *A. halys* groups and 5.56–46.44 μg/g in both *B. multicinctus* and *N. atra* groups. The mixture of venom and varespladib was administered to mice intraperitoneally. Deaths occurring within 24 h were recorded and the protective capacity was expressed as ED_50_ by the Spearman–Karber method [34].

### 4.6. Statistical Analysis

Data are expressed as the mean ± standard error of the mean (SEM). *T*-test and GraphPad Prism 6.01 were used to evaluate the statistical significance of differences. Statistical differences were considered significant when *p* values were <0.05.

## 5. Conclusions

Varespladib was effective in inhibiting svPLA_2_s. Hemorrhage and myonecrosis initiated by *D. acuts*, *A. halys*, *N. atra*, and *B. multicinctus* were significantly relieved by varespladib. Thus, varespladib has the potential for wide spectrum anti-venom drug development.

## Figures and Tables

**Table 1:** The ED_50_ of varespladib against challenge of four snake venoms.

Venom	Challenge Dose (μg)	ED_50_ (μg/g) (95% CI)	Standard Deviation
*D. acutus*	240	1.14 (0.86–1.50)	0.0608
*A. halys*	120	0.45 (0.33–0.62)	0.0699
*B. multicinctus*	12	15.23 (12.08–19.23)	0.0515
*N. atra*	28	22.09(17.31–28.19)	0.0540

## Abbreviations

CK	Creatine kinase
LDH1	Lactate dehydrogenase isoenzyme 1
AST	Aspartate transaminase
ALT	Alanine transaminase

## References

[1] Williams D., Gutiérrez J.M., Harrison R., Warrell D.A., White J., Winkel K.D., Gopalakrishnakone P. (2010). The Global Snake Bite Initiative: An antidote for snake bite. Lancet.

[2] Rägo L., Marroquin A.M., Nübling C.M., Sawyer J. (2015). Treating snake bites—A call for partnership. Lancet.

[3] Visser L.E., Kyei-Faried S., Belcher D.W., Geelhoed D.W., van Leeuwen J.S., van Roosmalen J. (2008). Failure of a new antivenom to treat Echis ocellatus snake bite in rural Ghana: The importance of quality surveillance. Trans. R. Soc. Trop. Med. Hyg..

[4] Calvete J.J., Arias A.S., Rodríguez Y., Quesada-Bernat S., Sánchez L.V., Chippaux J.P., Pla D., Gutiérrez J.M. (2016). Preclinical evaluation of three polyspecific antivenoms against the venom of Echis ocellatus: Neutralization of toxic activities and antivenomics. Toxicon.

[5] Laustsen A., Engmark M., Milbo C., Johannesen J., Lomonte B., Gutiérrez J., Lohse B. (2016). From Fangs to Pharmacology: The Future of Snakebite Envenoming Therapy. Curr. Pharm. Des..

[6] Herrera C., Macedo J.K., Feoli A., Escalante T., Rucavado A., Gutierrez J.M., Fox J.W. (2016). Muscle Tissue Damage Induced by the Venom of Bothrops asper: Identification of Early and Late Pathological Events through Proteomic Analysis. PLoS Negl. Trop. Dis..

[7] Pinho F.M.O., Yu L., Burdmann E.A. (2008). Snakebite-Induced Acute Kidney Injury in Latin America. Semin. Nephrol..

[8] Marsh N., Gattullo D., Pagliaro P., Losano G. (1997). The gaboon viper, Bitis gabonica: Hemorrhagic, metabolic, cardiovascular and clinical affects of the venom. Life Sci..

[9] Montecucco C., Gutiérrez J.M., Lomonte B. (2008). Cellular pathology induced by snake venom phospholipase A2 myotoxins and neurotoxins: Common aspects of their mechanisms of action. Cell. Mol. Life Sci..

[10] Fernández J., Vargas-Vargas N., Pla D., Sasa M., Rey-Suárez P., Sanz L., Gutiérrez J.M., Calvete J.J., Lomonte B. (2015). Snake Venomics of Micrurus Alleni and Micrurus Mosquitensis from the Caribbean Region of Costa Rica Reveals Two Divergent Compositional Patterns in New World Elapids. Toxicon.

[11] Gutiérrez J.M. (2014). Current challenges for confronting the public health problem of snakebite envenoming in Central America. J. Venom. Anim. Toxins Incl. Trop. Dis..

[12] Schaeffer T.H., Khatri V., Reifler L.M., Lavonas E.J. (2012). Incidence of immediate hypersensitivity reaction and serum sickness following administration of crotalidae polyvalent immune Fab antivenom: A meta-analysis. Acad. Emerg. Med..

[13] Schiermeier Q. (2015). Africa braced for snakebite crisis. Nature.

[14] Ranawaka U.K., Lalloo D.G., de Silva H.J. (2013). Neurotoxicity in Snakebite-The Limits of Our Knowledge. PLoS Negl. Trop. Dis..

[15] Gay C., Maruñak S., Teibler P., Leiva L., Acosta O. (2013). Effect of monospecific antibodies against baltergin in myotoxicity induced by bothrops alternatus venom from northeast of argentina. Role of metalloproteinases in muscle damage. Toxicon.

[16] Vaiyapuri S., Wagstaff S.C., Harrison R.A., Gibbins J.M., Hutchinson E.G. (2011). Evolutionary analysis of novel serine proteases in the Venom Gland transcriptome of Bitis gabonica rhinoceros. PLoS ONE.

[17] Francis B.R., Jorge Da Silva N., Seebart C., Casais E Silva L.L., Schmidt J.J., Kaiser I.I. (1997). Toxins isolated from the venom of the Brazilian coral snake (Micrurus frontalis frontalis) include hemorrhagic type phospholipases A2 and postsynaptic neurotoxins. Toxicon.

[18] Gutiérrez J.M., Ponce-Soto L.A., Marangoni S.L.B. (2008). Systemic and local myotoxicity induced by snake venom group II phospholipases A2: Comparison between crotoxin, crotoxin B and a Lys49 PLA2 homologue. Toxicon.

[19] De Carvalho N.D., Garcia R.C., Kleber Ferreira A., Rodrigo Batista D., Carlos Cassola A., Maria D., Lebrun I., Mendes Carneiro S., Castro Afeche S., Marcourakis T. (2014). Neurotoxicity of coral snake phospholipases A2 in cultured rat hippocampal neurons. Brain Res..

[20] Rudrammaji L.M., Machiah K.D., Kantha T.P., Gowda T.V. (2001). Role of catalytic function in the antiplatelet activity of phospholipase A2 cobra (Naja naja naja) venom. Mol. Cell. Biochem..

[21] Ferreira T., Camargo E.A., Ribela M.T.C.P., Damico D.C., Marangoni S., Antunes E., De Nucci G., Landucci E.C.T. (2009). Inflammatory oedema induced by Lachesis muta muta (Surucucu) venom and LmTX-I in the rat paw and dorsal skin. Toxicon.

[22] Garcia Denegri M.E., Maruñak S., Todaro J.S., Ponce-Soto L.A., Acosta O., Leiva L. (2014). Neutralisation of the pharmacological activities of Bothrops alternatus venom by anti-PLA2 IgGs. Toxicon.

[23] Snyder D.W., Bach N.J., Dillard R.D., Draheim S.E., Carlson D.G., Fox N., Roehm N.W., Armstrong C.T., Chang C.H., Hartley L.W. (1999). Pharmacology of LY315920/S-5920, [[3-(aminooxoacetyl)-2-ethyl-1-(phenylmethyl)-1*H*-indol-4-yl]oxy] acetate, a potent and selective secretory phospholipase A2 inhibitor: A new class of anti-inflammatory drugs, SPI. J. Pharmacol. Exp. Ther..

[24] Lewin M., Samuel S., Merkel J., Bickler P. (2016). Varespladib (LY315920) appears to be a potent, broad-spectrum, inhibitor of snake venom phospholipase A2 and a possible pre-referral treatment for envenomation. Toxins.

[25] Xiao H., Pan H., Liao K., Yang M., Huang C. (2017). Snake Venom PLA 2, a Promising Target for Broad-Spectrum Antivenom Drug Development. BioMed Res. Int..

[26] Bustillo S., García-Denegri M.E., Gay C., Van de Velde A.C., Acosta O., Angulo Y., Lomonte B., Gutiérrez J.M., Leiva L. (2015). Phospholipase A2 enhances the endothelial cell detachment effect of a snake venom metalloproteinase in the absence of catalysis. Chem.-Biol. Interact..

[27] Bustillo S., Gay C.C., Denegri M.E.G., Ponce-Soto L.A., de Kier Joffé E.B., Acosta O., Leiva L.C. (2012). Synergism between baltergin metalloproteinase and Ba SPII RP4 PLA2 from Bothrops alternatus venom on skeletal muscle (C2C12) cells. Toxicon.

[28] Xiong S., Luo Y., Zhong L., Xiao H., Pan H., Liao K., Yang M., Huang C. (2017). Investigation of the inhibitory potential of phospholipase A2inhibitor gamma from Sinonatrix annularis to snake envenomation. Toxicon.

[29] Pawlak A., Gil R.J., Kulawik T., Pronicki M., Karkucińska-Wieogonekckowska A., Szymańska-Deogonekbińska T., Gil K., Lagwinski N., Czarnowska E. (2012). Type of desmin expression in cardiomyocytes—A good marker of heart failure development in idiopathic dilated cardiomyopathy. J. Intern. Med..

[30] Pawlak A., Gil R.J., Grajkowska W., Nasierowska-Guttmejer A.M., Rzezak J., Kulawik T. (2013). Significance of low desmin expression in cardiomyocytes in patients with idiopathic dilated cardiomyopathy. Am. J. Cardiol..

[31] Rosenson R.S., Hislop C., Elliott M., Stasiv Y., Goulder M., Waters D. (2010). Effects of varespladib methyl on biomarkers and major cardiovascular events in acute coronary syndrome patients. J. Am. Coll. Cardiol..

[32] Le Z., Li X., Yuan P., Liu P., Huang C. (2015). Orthogonal optimization of prokaryotic expression of a natural snake venom phospholipase A2 inhibitor from Sinonatrix annularis. Toxicon.

[33] Liu D., Jiang K., Shu P. (2007). Snakes and snake toxins. Biotoxin Development and Utilization.

[34] Hamilton M., Russo R., Thurston R. (1977). Trimmed Spearman-Karber Method for Estimating Median Lethal Concentrations in Toxicity Bioassays. Environ. Sci. Technol..

