# Correction: Wang et al. Exploration of the Inhibitory Potential of Varespladib for Snakebite Envenomation. *Molecules* 2018, *23*, 391

**DOI:** 10.3390/molecules27165269

**Published:** 2022-08-18

**Authors:** Yiding Wang, Jing Zhang, Denghong Zhang, Huixiang Xiao, Shengwei Xiong, Chunhong Huang

**Affiliations:** School of Basic Medical Sciences, Nanchang University, Nanchang 330006, China

The authors would like to correct an error in the original publication [1]. This error is related to the picture misuse of *N. atra* of the varespladib- group in Figure 3a. We provide the correct Figure 3 below (the legend is unchanged):

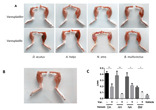



The authors apologize for any inconvenience caused and state that the scientific conclusions are unaffected. The original publication has also been updated. 

## References

[1] Wang Y., Zhang J., Zhang D., Xiao H., Xiong S., Huang C. (2018). Exploration of the Inhibitory Potential of Varespladib for Snakebite Envenomation. Molecules.

